# Presence of *Streptococcus mutans* strains harbouring the *cnm* gene correlates with dental caries status and IgA nephropathy conditions

**DOI:** 10.1038/srep36455

**Published:** 2016-11-04

**Authors:** Taro Misaki, Shuhei Naka, Rina Hatakeyama, Akiko Fukunaga, Ryota Nomura, Taisuke Isozaki, Kazuhiko Nakano

**Affiliations:** 1 Division of Nephrology, Seirei Hamamatsu General Hospital, Hamamatsu, Shizuoka 430-8558, Japan; 2 Department of Pediatric Dentistry, Division of Oral Infection and Disease Control, Osaka University Graduate School of Dentistry, Suita, Osaka, Japan; 3 Division of Dentistry, Seirei Hamamatsu General Hospital, Hamamatsu, Shizuoka, Japan

## Abstract

*Streptococcus mutans* is a major pathogen of human dental caries. Strains harbouring the *cnm* gene, which encodes Cnm, a collagen-binding protein, contribute to the development of several systemic diseases. In this study, we analysed *S. mutans* strains isolated from the oral cavity of immunoglobulin (Ig)A nephropathy (IgAN) patients to determine potential relationships between *cnm* and caries status as well as IgAN conditions. Saliva specimens were collected from 109 IgAN patients and the *cnm* status of isolated *S. mutans* strains was determined using PCR. In addition, the dental caries status (decayed, missing or filled teeth [DMFT] index) in patients who agreed to dental consultation (n = 49) was evaluated. The DMFT index and urinary protein levels in the *cnm*-positive group were significantly higher than those in the *cnm*-negative group (*p* < 0.05). Moreover, the urinary protein levels in the high DMFT (≥15) group were significantly higher than those in the low DMFT (<15) group (*p* < 0.05). Our results show that isolation of *cnm*-positive *S. mutans* strains from the oral cavity may be associated with urinary protein levels in IgAN patients, especially those with a high dental caries status.

Immunoglobulin (Ig)A nephropathy (IgAN) is the most frequent chronic glomerulonephritis in the world^1^,^2^. IgAN occurs at any age, most commonly with clinical onset in the second and third decades of life^3^. Approximately 30–40% of IgAN patients progress to end-stage kidney disease within 20 years^1^,^2^. However, there is no disease-targeted treatment for IgAN^4^ since pathogenesis of the disease remains unknown. IgAN patients often manifest deterioration of macroscopic haematuria in upper respiratory infections such as tonsillitis^3^. Some bacteria have been reported as the source of initiating antigens or involved in the pathogenesis of IgAN^5^,^6^,^7^,^8^,^9^, and *Haemophilus parainfluenzae* antigen and *Staphylococcus aureus* cell envelope antigens have been detected in renal tissue of IgAN patients^5^,^8^. In addition, periodontitis-related bacteria were also suggested to be associated with IgAN^10^. These reports suggest that infection may trigger IgAN.

*Streptococcus mutans*, a Gram-positive oral streptococcal species known to be a major pathogen of human dental caries^11^, is occasionally isolated from the blood of patients with infective endocarditis^12^. *S. mutans* strains harbouring the *cnm* gene, which encodes Cnm, a cell surface collagen-binding protein, exhibit binding ability to the extracellular matrix, which may be a possible virulence factor in infective endocarditis^13^. Furthermore, our recent studies suggest that *S. mutans* strains expressing Cnm on the cell surface may be associated with aggravated cerebral haemorrhaging^14^,^15^, non-alcoholic steatohepatitis^16^ and inflammatory bowel disease^11^. We occasionally encountered IgAN patients with inflammatory bowel disease in the clinical setting and an association between inflammatory bowel disease and IgAN has also been noted^17^. Thus, we hypothesized that the presence of *cnm*-positive *S. mutans* strains may affect IgAN. We previously showed that the rate of *cnm*-positive *S. mutans* strains isolated from the oral cavity was found to be significantly higher in IgAN patients than in non-diseased controls, suggesting that the presence of *cnm*-positive *S. mutans* strains in the oral cavity may influence IgAN severity^18^. Based upon the present study, we hypothesized that *cnm* status and dental caries status exacerbate urinary protein levels in IgAN patients.

## Results

### Characteristics of IgAN and non-diseased control groups

At presentation, IgAN patients and non-diseased control subjects exhibited similar age and sex distributions (Table 1). The rates of *S. mutans* isolation in the IgAN and control groups were similar, whereas *cnm*-positive strains were significantly more prevalent in the IgAN group than in the control group (*p* = 0.0465).

### Dental caries status between IgAN patients and non-diseased control subjects

Forty-nine of the 109 IgAN patients and 49 of the 61 non-diseased control subjects were evaluated for dental caries status. No significant differences were found between IgAN and control groups regarding missing teeth (MT) and filled teeth (FT) index values (Table 2). However, the IgAN group was significantly associated with higher values for decayed teeth (DT) index (*p* = 0.0058) and decayed, missing or filled teeth (DMFT) index values (*p* = 0.0074).

### Background differences between *S. mutans-*negative groups and *cnm*-positive and *cnm*-negative *S. mutans* groups

IgAN patients and non-diseased control subjects were divided into three groups each, an *S. mutans-*negative group and *cnm*-positive and *cnm*-negative *S. mutans* groups. No significant differences in gender were found between the *cnm*-positive *S. mutans* in IgAN patients group and other groups, whereas the *cnm*-positive *S. mutans* in IgAN patients group was significantly associated with % urinary protein 1+ or higher (Table 3). In addition, the association between % urinary protein 1+ or higher and *cnm*-positive *S. mutans* in IgAN patients remained significantly different in subsequent logistic regression analysis adjusted for age and gender (*p* = 0.0011) (Supplementary Table S1). Furthermore, the *cnm*-positive *S. mutans* in the IgAN patients group was also significantly associated with higher age, higher serum creatinine, lower estimated glomerular filtration rates (eGFR), % urinary occult blood 1+ or higher and higher DMFT index. However, these data did not remain significantly different in subsequent logistic regression analyses adjusted for age and gender (data not shown).

### Background differences in IgAN patients harbouring *cnm*-positive and *cnm*-negative *S. mutans* strains

**Figure 1:**
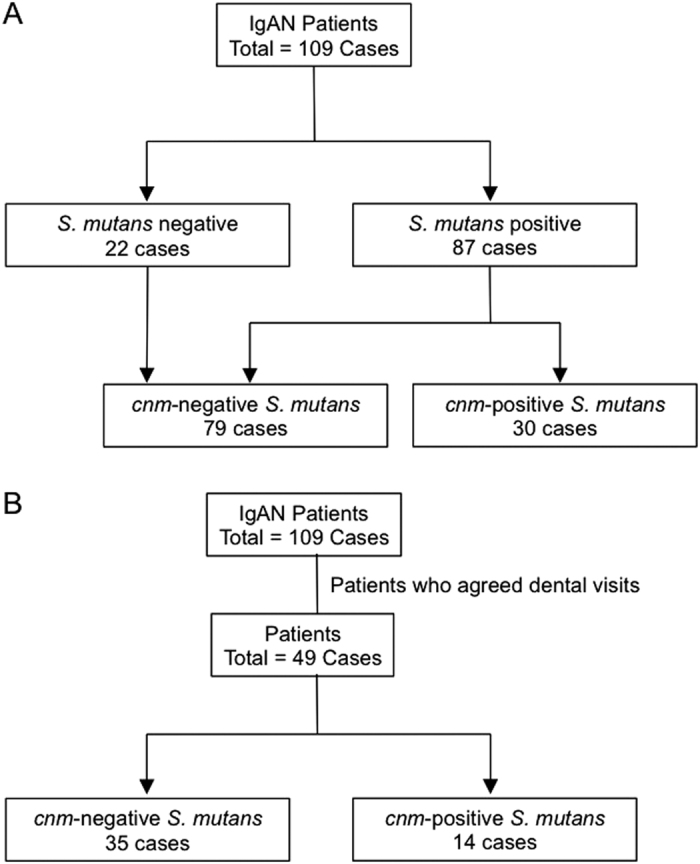
Patient enrolment. A total of 109 patients with IgAN were enrolled, among whom 30 showed positivity for the *cnm* gene (**A**). The dental caries status was evaluated in 49 patients, among whom 14 showed positivity for the *cnm* gene (**B**). IgAN: immunoglobulin (Ig)A nephropathy.

A total of 109 IgAN patients (mean age 43.8 ± 13.6 years, 62 males and 47 females) were analysed. *S. mutans* strains were isolated from 87 subjects with (n = 30) or without (n = 57) *cnm*-positive *S. mutans* strains in the oral cavity. Patients without *S. mutans* isolated strains (n = 22) were included in the *cnm*-negative *S. mutans* group (Fig. 1A). No significant differences were found between the *cnm*-positive *S. mutans* and *cnm*-negative *S. mutans* groups regarding sex, height, body weight, body mass index (BMI), serum creatinine, serum IgA, urinary red blood cell, anamnesis of tonsillectomy rate, anamnesis of steroid therapy rate or duration from kidney biopsy (Table 4).

The *cnm*-positive *S. mutans* group was significantly associated with higher age (*p* = 0.0026), higher systolic blood pressure (*p* = 0.0011), higher diastolic blood pressure (*p* = 0.0047), lower serum albumin (*p* = 0.0026), higher serum total cholesterol (*p* = 0.0258) and higher urinary protein levels (*p* = 0.0021) (Table 4). In addition, the association between urinary protein levels >0.5 g/gCr and *cnm*-positive *S. mutans* remained significantly different in subsequent logistic regression analysis adjusted for age and sex (*p* = 0.0147) (Supplementary Table S2). The *cnm*-positive *S. mutans* group was also significantly associated with lower eGFR (*p* = 0.0173) and higher renin-angiotensin system inhibitor (RAS-I) medication rates (*p* = 0.0444), although these data did not remain significantly different in subsequent logistic regression analysis adjusted for age and sex (data not shown).

### Dental caries status in the *cnm*-positive *S. mutans* and *cnm*-negative *S. mutans* groups in IgAN patients

Forty-nine of the 109 IgAN patients were evaluated for dental caries status (Fig. 1B), among whom 14 subjects showed positivity for the *cnm* gene. No significant differences were found between *cnm*-positive *S. mutans* and *cnm*-negative groups regarding the DT and MT indices. However, the *cnm*-positive *S. mutans* group was significantly associated with higher values for the FT (*p* = 0.0017) and DMFT indices (*p* = 0.0400) (Table 5). In addition, the association between *cnm*-positive *S. mutans* and higher DMFT index values remained significantly different in subsequent logistic regression analysis adjusted for age and sex (*p* = 0.0468) (Supplementary Table S3).

### Clinical differences between high and low DMFT index groups in IgAN patients

**Figure 2:**
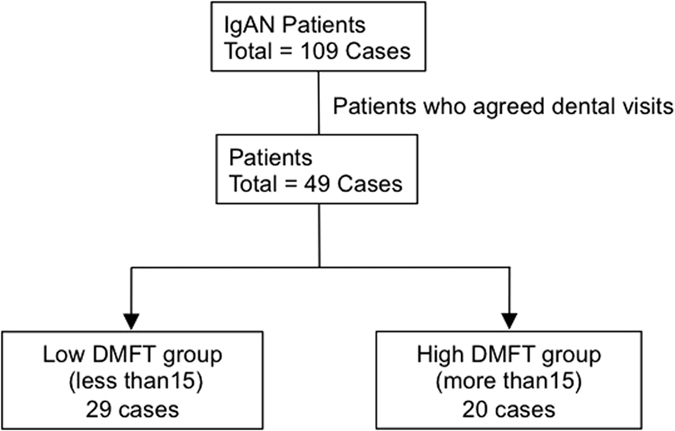
Dental caries status of IgAN patients. Among 49 subjects, 20 had DMFT values of 15 or greater and were designated as the ‘high DMFT group’. IgAN: immunoglobulin (Ig)A nephropathy, DMFT: decayed, missing or filled teeth.

A total of 49 IgAN patients (mean age 44.4 ± 13.9 years, 27 males and 22 females) were analysed (Fig. 2), among whom 20 subjects showed DMFT index values of 15 or greater (designated as the ‘high DMFT group’). The remaining 29 subjects with DMFT index values lower than 15 were designated as the ‘low DMFT group’. There were no significant differences found between the high and low DMFT groups regarding sex, height, body weight, BMI, diastolic blood pressure, serum albumin, serum IgA, urinary red blood cell, RAS-I medication rate, anamnesis of tonsillectomy rate, anamnesis of steroid therapy rate or duration from kidney biopsy. However, the high DMFT group was significantly associated with higher age (*p* = 0.0108), higher urinary protein levels (*p* = 0.0246), higher serum creatinine (*p* = 0.0026) and lower eGFR (*p* = 0.0012) (Table 6). In addition, the association between urinary protein levels >0.5 g/gCr and higher DMFT index values remained significantly different in subsequent logistic regression analysis adjusted for age and sex (*p* = 0.0076) (Supplementary Table S4). Furthermore, the high DMFT group was also significantly associated with higher systolic blood pressure (*p* = 0.0432), although these data did not remain significantly different in subsequent logistic regression analysis adjusted for age and gender (data not shown).

### *cnm*-positivity and the DMFT index are important factors for proteinuria in IgAN patients

**Figure 3:**
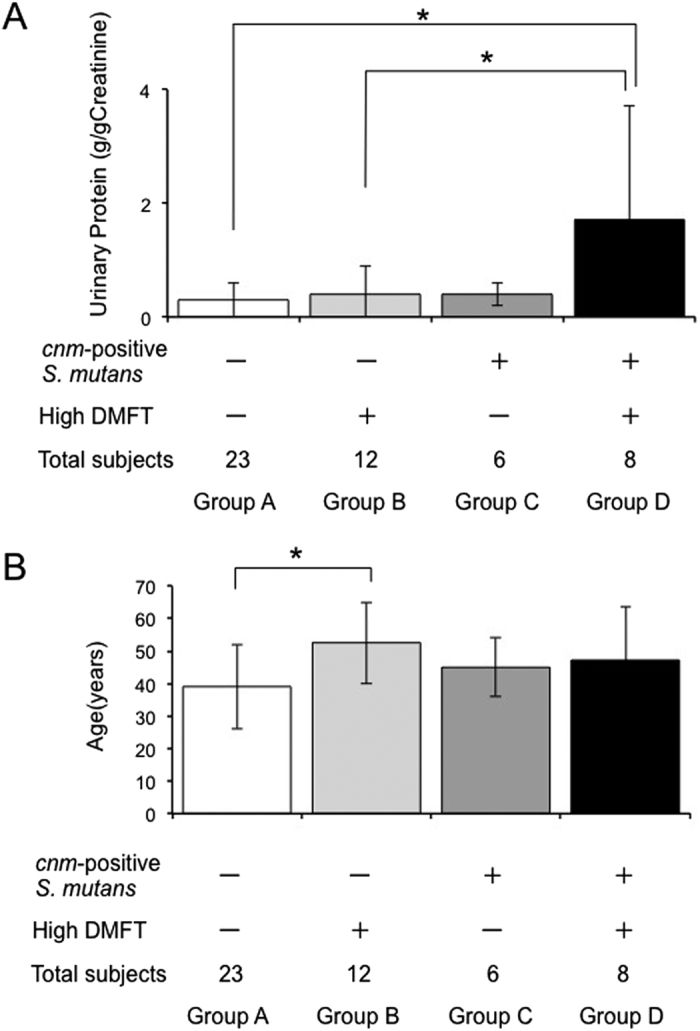
Relationship between proteinuria and *cnm* positivity and DMFT status. (**A**) Group D was significantly associated with higher urinary protein levels than all other groups (*p < 0.05). (**B**) Group D was not significantly associated with higher age than all other groups. A: *cnm*-negative *S. mutans* and low DMFT group (n = 23), B: *cnm*-negative *S. mutans* and high DMFT group (n = 12), (**C**) *cnm*-positive *S. mutans* and low DMFT group (n = 6) and (**D**) *cnm*-positive *S. mutans* and high DMFT group (n = 8). DMFT: decayed, missing or filled teeth. The high DMFT group was designated as patients having a DMFT index of 15 or greater. The low DMFT group was designated as patients having a DMFT index of lower than 15.

A total of 49 IgAN patients who underwent evaluation of dental caries status were divided into four groups according to *cnm* positivity and DMFT index value as follows: A: *cnm*-negative *S. mutans* and low DMFT group (n = 23), B: *cnm*-negative *S. mutans* and high DMFT group (n = 12), C: *cnm*-positive *S. mutans* and low DMFT group (n = 6) and D: *cnm*-positive *S. mutans* and high DMFT group (n = 8). Group D (1.7 ± 2.0 g/g creatinine) was significantly associated with higher urinary protein levels compared with all other groups (Group A: 0.3 ± 0.3 g/g creatinine, *p* = 0.0001; Group B: 0.4 ± 0.5 g/g creatinine, *p* = 0.0013; and Group C: 0.4 ± 0.2 g/g creatinine, *p* = 0.0047) (Fig. 3A), although Group D was not significantly associated with higher age compared with all other groups (Fig. 3B). In addition, Group B was significantly associated with higher age compared with Group A (*p* = 0.0059).

## Discussion

To our knowledge, this is the first study demonstrating that *cnm* positivity and dental caries status are associated with urinary protein levels in IgAN patients. Although several studies using *in vivo* approaches in the 1980s and 1990s hypothesized a correlation between *S. mutans* and nephritis^19^,^20^, there have been no reports regarding comparable human data. There are currently no established disease-targeted treatments for IgAN because the precise pathogenic mechanism involved remains unclear^4^. Therefore, we believe that the strong correlation between *S. mutans* and IgAN presented in this study could provide important information for future research regarding IgAN therapy.

The present study clearly demonstrated that IgAN patients harbouring *cnm*-positive *S. mutans* strains in the oral cavity showed significantly higher DMFT index values and urinary protein levels compared with the *cnm*-negative *S. mutans* group. In addition, the *cnm*-positive *S. mutans* group showed significantly lower serum albumin levels, higher serum total cholesterol levels and higher blood pressure than the *cnm*-negative group. In general, proteinuria causes hypoalbuminemia and hypercholesterolemia, both of which are sometimes seen in IgAN^21^. Hypertension in IgAN is also associated with renal damage^22^. Therefore, it is reasonable to consider that these clinical data also strengthen our hypothesis, in which *cnm*-positive *S. mutans* strains present in the oral cavity aggravate IgAN symptoms. However, the precise mechanism by which the presence of *cnm*-positive *S. mutans* strains in the oral cavity influences serum albumin, cholesterol and blood pressure in IgAN patients remains to be elucidated. The present study also demonstrated that *cnm*-positive strains were significantly more prevalent in the IgAN group than in the non-diseased control group, and the prevalence of dental caries was significantly higher in the IgAN group than in the non-diseased control group. These data suggest that IgAN patients may have more dental caries than non-diseased controls, which may exacerbate IgAN conditions. However, other factors such as socioeconomic status may be involved and should be analysed in future studies.

The concept of the ‘kidney-gut axis’ has recently gained attention^23^,^24^ and there is increasing clinical evidence that patients with chronic kidney disease have a distinctly dysbiotic intestinal bacterial community (termed ‘gut microbiota’). This, in turn, drives a cascade of metabolic abnormalities, including uremic toxin production, inflammation and immunosuppression, which ultimately promote progressive kidney failure and cardiovascular disease^23^. Marked differences in gut microbiota composition were found between healthy controls and patients with end-stage renal disease using phylogenetic microarrays^25^. Some researchers have also suggested a novel hypothesis for the ‘intestine-kidney connection’ in IgAN^17^,^26^. A defective immune tolerance might favour an abnormal response to microbiota with alterations of the intestinal barrier, including increased alimentary antigens and bacterial toxins absorption, triggering mucosal-associated lymphoid tissue activation and subclinical intestinal inflammation^26^. This can produce an abnormal response to alimentary antigens or commensal microbes with synthesis of aberrantly glycosylated polymeric IgA1, which eventually enters the circulation with renal deposit formation^26^. Because *S. mutans* is a major pathogen of dental caries, our data provide evidence for a new concept of ‘oral-kidney association’. Future experimental approaches should focus on determining how oral infectious pathogens induce or aggravate IgAN. We believe that the strong correlation between *S. mutans* and IgAN shown in the present study could provide a new breakthrough for future prevention and intervention for IgAN.

Interestingly, we found that a high DMFT value (designated as values of 15 or greater) was associated with high urinary protein levels and low renal function in IgAN patients, suggesting that IgAN patients who have elevated dental caries experience could have higher urinary protein levels and lower renal function. When analysing possible factors that influence proteinuria (i.e., *cnm* positivity and high DMFT index value), both factors were clearly important. Because it is true that older subjects tend to have higher DMFT index values, it is possible that age itself may influence IgAN severity. However, the *cnm*-positive and high DMFT group was not significantly associated with higher age compared with all other groups (Fig. 3B). We also performed subsequent logistic regression analyses adjusted for age and sex, and our findings led us to consider that patients with these two factors may exhibit severe IgAN aggravation.

Unfortunately, the present study did not demonstrate sufficient evidence to determine the possible mechanism by which *S. mutans* strains, especially *cnm*-positive strains, contribute to the development of IgAN. However, we speculate that this may involve the following hypotheses. First, frequent, repeated *cnm*-positive *S. mutans* immunoreactions with IgA in mucosal tissues of the oral cavity might induce a glycosylation defect in serum IgA1 molecules, which play an important role in the pathogenesis of IgAN. Many studies have revealed that a glycosylation deficiency in IgA1 molecules, usually with reduced galactose and sialic acid content but increased exposure of N-acetylgalactosamine, is a primary characteristic of IgAN^4^,^27^,^28^. This pattern of glycosylation mostly affects polymeric IgA1 produced in mucosal tissues^4^. Second, *cnm*-positive *S. mutans* strains or Cnm antigen itself may bind to mesangial cells, endothelial cells or glomerular basement membrane directly and induce mesangial cell proliferation and extracellular matrix expansion or endocapillary damage transiently. In general, severe dental caries results in the destruction of enamel and/or dentine on the tooth surface, enabling *cnm*-positive *S. mutans* access to the bloodstream. Further studies are required to elucidate the detailed mechanisms involved.

It is unclear why age appeared to be a significant factor in the *cnm*-positive IgAN group compared with the *cnm-*negative IgAN group. Generally, *S. mutans* is acquired in the oral cavity from mother’s saliva in early childhood, and *cnm*-positive *S. mutans* strains were demonstrated to be transmitted from mothers to their children^29^. It is possible that *cnm*-positive strains could be transmitted from those in close contact via saliva in adults. Although there are no reports investigating age as a factor in the distribution rates of *cnm*-positive strains, it is reasonable to speculate that the distribution rate could increase in older-aged subjects. In fact, it has been suggested that the positivity rate of *cnm*-positive *S. mutans* in older people (mean 70.3 years) was relatively higher (36.7%) than in the general population^30^. We collected specimens from outpatients in our hospital and thus selection bias may exist. For instance, stable IgAN patients were not included in these studies, but rather aged, severe long-term sufferers. However, we attempted to avoid such bias by performing logistic regression analyses adjusted for age.

It should be noted that there are some limitations associated with this study. First, we only showed that *cnm* positivity and dental caries status were associated with increased urinary protein levels in IgAN patients. However, it is possible that IgAN patients are susceptible to *cnm*-positive *S. mutans* strains. Whether persistent *cnm*-positive *S. mutans* infection can induce IgAN must be confirmed using experimental rodent models. Second, it remains unclear whether *S. mutans* directly contributes to the development of IgAN. Ultimately, the precise pathogenic mechanism of IgAN induction must be determined. Third, we were unable to perform additional analyses to evaluate potential confounders, such as diet and socioeconomic status, in the present study. Fourth, the *cnm*-positive *S. mutans* rate in other kidney diseases remains unclear. Finally, this study enrolled a relatively small number of IgAN patients from a single ethnic group. Prospective and larger studies should be designed to confirm our results.

## Methods

### Subjects and clinical data

The subjects were IgAN patients who are outpatients of Seirei Hamamatsu General Hospital, Hamamatsu, Japan. These patients were diagnosed with IgAN by previous renal biopsies. The histological diagnosis was made based on light microscopy and immunohistochemistry findings. Patients who had secondary IgAN diseases, such as IgA vasculitis (Henoch-Schonlein purpura nephritis) or lupus nephritis, were excluded from this study. IgAN patients undergoing steroid or immunosuppression therapy were also excluded from this study. The clinical data (urinary protein excretion and urinary occult blood by dipsticks, urinary protein excretion/urinary creatinine excretion, urinary red blood cell, serum creatinine, estimated glomerular filtration rate (eGFR), serum IgA, serum albumin, total cholesterol, blood pressure, height and body weight) were collected at the time of informed consent.

Age-matched healthy subjects were included as the non-diseased control group. All subjects were confirmed to have normal kidney function (serum Cr < 1.2 mg/dl, no proteinuria and no haematuria), were not taking regular medications and had no history of any other diseases, such as diabetes mellitus, hypertension, stroke, heart failure, rheumatoid arthritis, liver disease, gastrointestinal disease or anaemia.

### Isolation of *S. mutans* strains from saliva specimens

Non-stimulated expectorated whole saliva specimens were collected from a total of 109 IgAN patients and 61 saliva specimens from control subjects from October 2013 to March 2016 using a sterile plastic tube and stored at −20 °C. *S. mutans* strains were isolated within a month after collection and confirmed as previously described^31^. Briefly, saliva specimens (approximately 1 ml from each subject) were diluted and streaked onto Mitis Salivarius agar plates (Difco Laboratories, Detroit, MI, USA) containing bacitracin (0.2 U/ml; Sigma-Aldrich, St. Louis, MO, USA) and 15% (wt/vol) sucrose and incubated at 37 °C for 2 days anaerobically. Five colonies from each plate were randomly selected on the basis of rough colony morphology and stocked. To differentiate *S. mutans* from *Streptococcus sobrinus*, colony morphology on agar plates was initially evaluated. Rough and smooth colonies were considered to be *S. mutans* and *S. sobrinus*, respectively. *S. mutans* strains were then confirmed via PCR using glucosyltransferase (*gtf*)-based primer sets as described below.

### Bacterial DNA extraction and PCR detection of *cnm*

Strains were grown in brain heart infusion medium (Difco) at 37 °C for 18 hours, and genomic DNA was then extracted using conventional methods. Then, PCR was performed to confirm that these strains were *S. mutans* using an *S. mutans*-specific primer set (Forward: 5′–GGC ACC ACA ACA TTG GGA AGC TCA GTT–3′, Reverse: 5′–GGA ATG GCC GCT AAG TCA ACA GGA T–3′), as described previously^14^. The *S. mutans*-specific primer set for PCR was designed based on the sequence of glucosyltransferase *gtf* gene to differentiate *S. mutans* from *S. sobrinus*. PCR was also performed to detect *cnm*-positive strains using a *cnm*-specific primer set (Forward: 5′–GAC AAA GAA ATG AAA GAT GT–3′, Reverse: 5′–GCA AAG ACT CTT GTC CCT GC–3′), as described previously^14^.

### Evaluation of dental caries status

The dental caries status of 49 IgAN patients and 49 non-diseased control subjects who provided signed informed consent for dental consultation was evaluated by a single dentist (AF) using conventional methods at the Department of Dentistry, Seirei Hamamatsu General Hospital. The clinical dental examination included the number of total teeth present and the presence of decayed teeth (DT), missing teeth (MT) and filled teeth (FT). Next, the DMFT index with the total numbers of D, M and F was calculated as described previously^32^.

### Ethics regarding subjects

This study was conducted in full adherence with the Declaration of Helsinki (64th WMA General Assembly, Fortaleza, Brazil, 2013), and study protocols were approved by the Ethics Committee of Seirei Hamamatsu General Hospital (approval no. 1807) and Osaka University Graduate School of Dentistry (approval no. H25-E24). All subjects were informed of the protocols and gave their written consent prior to participating in the study.

### Statistical analysis

All results are expressed as the mean ± standard deviation (SD). When a significant difference was found, further statistical analysis was performed using Fisher’s PLSD test, the Mann–Whitney U test or Fisher’s exact test and logistic regression analysis between groups. A two-tailed p-value of 0.05 was considered significant. Statistical analyses were performed using STATVIEW software (SAS Institute Inc., Cary, NC, USA) and SAS 9.1 software (SAS Institute Inc.).

## Additional Information

**How to cite this article**: Misaki, T. *et al*. Presence of *Streptococcus mutans* strains harbouring the *cnm* gene correlates with dental caries status and IgA nephropathy conditions. *Sci. Rep.* **6**, 36455; doi: 10.1038/srep36455 (2016).

**Publisher’s note:** Springer Nature remains neutral with regard to jurisdictional claims in published maps and institutional affiliations.

## Supplementary Material

Supplementary Information

## Figures and Tables

**Table 1:** Characteristics of IgAN and non-diseased control groups.

Characteristics	Control subjects (n = 61)	IgAN patients (n = 109)	p-value
Age (yr; mean ± SD)	42.1 ± 10.6	43.8 ± 13.6	0.4073
Gender (M/F)	33/28	62/47	0.7279
*S. mutans* isolated/total subjects (%)	80.3	79.8	0.9367
***cnm*****-positive*****S. mutans*****/total subjects (%)**	**11.5**	**27.5**	**0.0465**
***cnm*****-positive*****S. mutans*****/total** ***S. mutans*****isolated subjects (%)**	**14.2**	**34.4**	**0.0486**

Bold values indicate statistical significance at *p* < 0.05.

**Table 2:** Dental caries status between IgAN patients and control subjects.

Characteristics	Control subjects (n = 49)	IgAN patients (n = 49)	p-value
**DT index**	**0.3 ± 0.5**	**1.2 ± 2.3**	**0.0058**
MT index	0.7 ± 3.4	1.8 ± 4.8	0.2616
FT index	8.6 ± 5.4	10.1 ± 6.2	0.1849
**DMFT index**	**9.6 ± 5.9**	**13.1 ± 6.9**	**0.0074**

Bold values indicate statistical significance at *p* < 0.05.

**Table 3:** Background differences between *S. mutans* negative groups and *cnm*-positive and *cnm*-negative *S. mutans* groups.

Characteristics	Control subjects			IgAN patients		
	*S. mutans* negative (n = 12)	*S. mutans* positive		*S. mutans* negative (n = 22)	*S. mutans* positive	
		*cnm*-negative (n = 42)	*cnm*-positive (n = 7)		*cnm*-negative (n = 57)	*cnm*-positive (n = 30)
Age (yr; mean ± SD)	**41.2 ± 8.8 p = 0.0357**	**41.8 ± 11.2 p = 0.0057**	45.6 ± 10.3 p = 0.3836	**41.3 ± 14.7 p = 0.0122**	**41.5 ± 12.6 p = 0.0023**	**50.1 ± 13.1**
Gender (M/F)	7/5 p = 0.7701	24/18 p = 0.7504	2/5 p = 0.2398	15/7 p = 0.2917	31/26 p = 0.9258	16/14
Serum creatinine (mg/dl; mean ± SD)	0.8 ± 0.2 p = 0.1247	**0.7 ± 0.2 p = 0.0088**	0.7 ± 0.2 p = 0.1410	1.2 ± 1.2 p = 0.6026	0.9 ± 0.4 p = 0.1953	**1.1 ± 0.6**
eGFR (ml/min; mean ± SD)	**82.4 ± 15.6 p = 0.0032**	**89.4 ± 13.2 p < 0.0001**	77.5 ± 7.0 p = 0.0532	70.6 ± 27.7 p = 0.0845	**73.6 ± 22.8 p = 0.0057**	**60.5 ± 22.5**
% Urinary Protein 1 + or higher	**0 p < 0.0001**	**0 p < 0.0001**	**0 p < 0.0001**	**18.2 p < 0.0001**	**47.4 p = 0.0271**	**66.7**
% Urinary occult blood 1 + or higher	**0 p = 0.0004**	**0 p < 0.0001**	**0 p = 0.0036**	**22.7 p = 0.0248**	**29.8 p = 0.0491**	**46.7**
DMFT index (mean ± SD)	**3.8 ± 3.9 p = 0.0002 (n = 5)**	**10.1 ± 6.1 p = 0.0020 (n = 38)**	11.2 ± 3.3 p = 0.0942 (n = 6)	**10.5 ± 8.2 p = 0.0239 (n = 11)**	12.5 ± 6.8 p = 0.0698 (n = 24)	**16.3 ± 4.8 (n = 14)**

The p-values in each column indicate comparison with the *cnm*-positive *S. mutans* in IgAN patients group.

Bold values indicate statistical significance at *p* < 0.05.

**Table 4:** Background differences between *cnm*-positive and *cnm*-negative *S. mutans* groups in IgAN patients.

Characteristics	Total (n = 109)	*cnm*-negative *S. mutans* (n = 79)	*cnm*-positive *S. mutans* (n = 30)	p-value
**Age (yr; mean ± SD)**	**43.8 ± 13.6**	**41.4 ± 13.1**	**50.1 ± 13.1**	**0.0026**
Gender (M/F)	62/47	46/33	16/14	0.6486
Height (cm; mean ± SD)	164.1 ± 8.5	165.0 ± 8.8	161.8 ± 7.4	0.0867
Body weight (kg; mean ± SD)	60.6 ± 12.1	60.9 ± 10.7	59.9 ± 15.4	0.6976
BMI (kg/m^2^;mean ± SD)	22.3 ± 3.6	22.2 ± 3.2	22.7 ± 4.6	0.5834
**Systolic blood pressure (mmHg; mean ± SD)**	**123.8 ± 16.1**	**120.8 ± 14.4**	**131.8 ± 17.8**	**0.0011**
**Diastolic blood pressure (mmHg; mean ± SD)**	**78.2 ± 11.9**	**76.3 ± 11.5**	**83.4 ± 11.4**	**0.0047**
**Serum albumin (g/dl; mean ± SD)**	**4.2 ± 0.5**	**4.2 ± 0.4**	**4.0 ± 0.5**	**0.0026**
**Serum total cholesterol (mg/dl; mean ± SD)**	**193.3 ± 36.6**	**188.4 ± 31.3**	**205.9 ± 45.8**	**0.0258**
Serum creatinine (mg/dl; mean ± SD)	1.0 ± 0.7	1.0 ± 0.7	1.1 ± 0.6	0.5161
**eGFR (ml/min/1.73 m**^**2**^**; mean ± SD)**	**69.4 ± 24.2**	**72.8 ± 24.1**	**60.5 ± 22.5**	**0.0173**
Serum IgA (mg/dl; mean ± SD)	291.3 ± 112.6	282.8 ± 113.3	312.9 ± 109.9	0.2242
**Urinary protein (g/gCr; mean ± SD)**	**0.7 ± 1.7**	**0.4 ± 0.9**	**1.5 ± 2.8**	**0.0021**
Urinary red blood cell (/HPF; mean ± SD)	5.3 ± 14.7	4.0 ± 12.1	8.7 ± 19.9	0.1319
**RAS-I medication rate (%)**	**76.1**	**70.9**	**90**	**0.0444**
Anamnesis of tonsillectomy rate (%)	40.4	39.2	43.3	0.7005
Anamnesis of steroid therapy rate (%)	77.1	72.2	90	0.0723
Duration from kidney biopsy (month; mean ± SD)	92.5 ± 68.5	91.7 ± 68.3	94.7 ± 70.3	0.8398

BMI: body mass index, eGFR: estimated glomerular filtration rate and RAS-I: renin-angiotensin system inhibitor. Bold values indicate statistical significance at *p* < 0.05.

**Table 5:** Dental caries status between *cnm*-positive and *cnm*-negative *S. mutans* groups in IgAN patients.

Characteristics	Total (n = 49)	*cnm*-negative *S. mutans* (n = 35)	*cnm*-positive *S. mutans* (n = 14)	p-value
DT index (mean ± SD)	1.2 ± 2.3	0.6 ± 1.3	1.5 ± 2.6	0.2735
MT index (mean ± SD)	1.8 ± 4.8	2.2 ± 5.5	0.9 ± 1.7	0.3848
**FT index (mean ± SD)**	**10.1 ± 6.2**	**8.4 ± 6.0**	**14.4 ± 4.5**	**0.0017**
**DMFT index (mean ± SD)**	**13.1 ± 6.9**	**11.9 ± 7.2**	**16.3 ± 4.8**	**0.0400**

DT: decayed teeth, MT: missing teeth, FT: filled teeth and DMFT: decayed, missing or filled teeth. Bold values indicate statistical significance at *p* < 0.05.

**Table 6:** Clinical data between the high and low DMFT index groups in IgAN patients.

Characteristics	Total (n = 49)	Low DMFT (less than 15) (n = 29)	High DMFT (15 and more) (n = 20)	p-value
**Age (yr; mean ± SD)**	**44.4 ± 13.9**	**40.3 ± 12.4**	**50.4 ± 14.1**	**0.0108**
Gender (M/F)	27/22	16/13	11/9	0.9907
Height (cm; mean ± SD)	163.3 ± 7.9	162.9 ± 8.1	163.9 ± 7.8	0.7027
Body weight (kg; mean ± SD)	62.1 ± 13.4	61.7 ± 12.2	62.7 ± 15.2	0.7946
BMI (kg/m^2^; mean ± SD)	23.0 ± 4.4	22.8 ± 3.6	23.3 ± 5.3	0.6846
**Systolic blood pressure (mmHg; mean ± SD)**	**126.0 ± 16.3**	**122.1 ± 16.6**	**131.7 ± 14.6**	**0.0432**
Diastolic blood pressure (mmHg; mean ± SD)	78.8 ± 11.5	76.6 ± 12.9	82.0 ± 8.5	0.1065
Serum albumin (g/dl; mean ± SD)	4.2 ± 0.4	4.3 ± 0.4	4.1 ± 0.4	0.1139
**Serum creatinine (mg/dl; mean ± SD)**	**0.9 ± 0.3**	**0.8 ± 0.2**	**1.1 ± 0.4**	**0.0026**
**eGFR (ml/min/1.73 m**^**2**^**; mean ± SD)**	**70.7 ± 22.3**	**78.9 ± 18.5**	**58.7 ± 22.4**	**0.0012**
IgA (mg/dl; mean ± SD)	306.9 ± 115.3	309.6 ± 125.6	302.9 ± 102.0	0.8479
**Urinary protein (g/gCr; mean ± SD)**	**0.5 ± 1.0**	**0.3 ± 0.3**	**0.9 ± 1.4**	**0.0246**
Urinary red blood cell (/HPF; mean ± SD)	7.0 ± 16.4	7.9 ± 19.2	5.6 ± 11.6	0.6305
RAS-I medication rate (%)	79.6	75.9	85	0.4459
Anamnesis of tonsillectomy rate (%)	44.9	44.8	45.0	0.9907
Anamnesis of steroid therapy rate (%)	75.5	75.9	75.0	0.9464
Duration from kidney biopsy (month;mean ± SD)	73.4 ± 55.1	65.9 ± 46.9	84.3 ± 64.8	0.2554

BMI: body mass index, eGFR: estimated glomerular filtration rate, RAS-I: renin-angiotensin system inhibitor. Bold values indicate statistical significance at *p* < 0.05.
