# Supplementary material for: Presence of Streptococcus mutans strains harbouring the cnm gene correlates with dental caries status and IgA nephropathy conditions
Source: Sci Rep. 2016 Nov 4;6:36455. doi: 10.1038/srep36455 (PMC5095553; doi:10.1038/srep36455)
Supplement: Supplementary Information [file srep36455-s1.doc]

**Supplementary Information**

**Presence of *Streptococcus mutans* strains harbouring the *cnm* gene correlates with dental caries status and IgA nephropathy conditions**

Taro Misaki^1^, Shuhei Naka^2^, Rina Hatakeyama^2^, Akiko Fukunaga^3^, Ryota Nomura^2^, Taisuke Isozaki^1^ and Kazuhiko Nakano^2,*^

^1^Division of Nephrology, Seirei Hamamatsu General Hospital, Hamamatsu, Shizuoka 430-8558, Japan, ^2^ Department of Pediatric Dentistry, Division of Oral Infection and Disease Control, Osaka University Graduate School of Dentistry, Suita, Osaka, Japan, ^3^Division of Dentistry, Seirei Hamamatsu General Hospital, Hamamatsu, Shizuoka, Japan

**Correspondence should be addressed to:**

Kazuhiko Nakano, DDS, PhD., Department of Pediatric Dentistry, Osaka University Graduate School of Dentistry, 1-8 Yamada-oka, Suita, Osaka 565-0871, Japan.

E-mail: [nakano@dent.osaka-u.ac.jp](mailto:nakano@dent.osaka-u.ac.jp); Fax: +81-6-6879-2965.

**Supplementary information includes:**

Supplementary Tables S1 to S4.

**Supplementary Tables**

**Supplementary Table S1: Association between % urinary protein 1+ or higher and *cnm*-positive *S. mutans* in IgAN patients.**

| **Variables** | | | **Odds ratio (95% confidence interval)** | **p-value** |
| --- | --- | --- | --- | --- |
| Age | | | 1.005 (0.974–1.038) | 0.7557 |
| Sex (Male) | | | 1.919 (0.824–4.468) | 0.1308 |
| IgAN patients | *S. mutans* negative | | Reference |  |
|  | *S. mutans* positive | ***cnm*-negative** | **4.564 (1.341**–**15.539)** | **0.0151** |
|  |  | ***cnm*-positive** | **10.011 (2.510–39.924)** | **0.0011** |
| Control subjects | *S. mutans* negative | | 8.100 × 10^-9^ (0.000) | 0.9979 |
|  | *S. mutans* positive | *cnm*-negative | 8.080 × 10^-9^ (0.000) | 0.9960 |
|  |  | *cnm*-positive | 9.580 × 10^-9^ (0.000) | 0.9984 |

Additive logistic regression models, adjusted for age and sex, were used for these analyses. The association between % urinary protein 1+ or higher and *cnm*-positive *S. mutans* in IgAN patients remained significantly different in subsequent logistic regression analysis adjusted for age and sex. Bold values indicate statistical significance at *p*<0.05. Statistical analyses were performed using STATVIEW software (SAS Institute Inc., Cary, NC, USA) and SAS 9.1 software (SAS Institute Inc.).

**Supplementary Table S2: Association between urinary protein levels >0.5 g/gCr and *cnm*-positive *S. mutans*.**

| **Variables** | **Odds ratio (95% confidence interval)** | **p-value** |
| --- | --- | --- |
| Age | 1.017 (0.982-1.053) | 0.3420 |
| Sex (Male) | 1.066 (0.421-2.697) | 0.8930 |
| ***cnm*-positive *S. mutans*** | **3.281 (1.263-8.524)** | **0.0147** |

Additive logistic regression models, adjusted for age and sex, were used for these analyses. The association between urinary protein levels >0.5 g/gCr and *cnm*-positive *S. mutans* remained significantly different in subsequent logistic regression analysis adjusted for age and sex. Bold value indicates statistical significance at *p*<0.05. Statistical analyses were performed using STATVIEW software (SAS Institute Inc., Cary, NC, USA) and SAS 9.1 software (SAS Institute Inc.).

**Supplemental Table S3: Association between *cnm*-positive *S. mutans* and DMFT index.**

| **Variables** | **Odds ratio (95% confidence interval)** | **p-value** |
| --- | --- | --- |
| Age | 0.981 (0.924-1.042) | 0.5418 |
| Sex(Male) | 0.815 (0.204-3.249) | 0.7716 |
| **DMFT index** | **1.136 (1.002-1.289)** | **0.0468** |

Additive logistic regression models, adjusted for age and sex, were used for the analyses.

The association between *cnm*-positive *S. mutans* and higher DMFT index value remained significantly different in subsequent logistic regression analysis adjusted for age and sex. Bold value indicates statistical significance at *p*<0.05. Statistical analyses were performed using STATVIEW software (SAS Institute Inc., Cary, NC, USA) and SAS 9.1 software (SAS Institute Inc.).

**Supplementary Table S4: Association between urinary protein levels >0.5 g/gCr and DMFT index.**

| **Variables** | **Odds ratio (95% confidence interval)** | **p-value** |
| --- | --- | --- |
| Age | 0.963 (0.900-1.031) | 0.2787 |
| Sex(Male) | 0.689 (0.152-3.121) | 0.6287 |
| **DMFT index** | **1.226 (1.056-1.423)** | **0.0076** |

Additive logistic regression models, adjusted for age and sex, were used for the analyses.

The association between urinary protein levels >0.5 g/gCr and higher DMFT index value remained significantly different in subsequent logistic regression analysis adjusted for age and sex. Bold value indicates statistical significance at *p*<0.05. Statistical analyses were performed using STATVIEW software (SAS Institute Inc., Cary, NC, USA) and SAS 9.1 software (SAS Institute Inc.).
